# Modification of Chitosan Membranes via Methane Ion Beam

**DOI:** 10.3390/molecules25102292

**Published:** 2020-05-13

**Authors:** Nasim Gholami, Babak Jaleh, Reza Golbedaghi, Majid Mojtahedzadeh Larijani, Pikul Wanichapichart, Mahmoud Nasrollahzadeh, Rajender S. Varma

**Affiliations:** 1 Physics Department, Bu-Ali Sina University, Hamedan 65174, Iran; n.gholami3253@yahoo.com; 2 Chemistry Department, Payame Noor University, Tehran 19395–4697, Iran; golbedaghi82@gmail.com; 3 Physics and Accelerators Research School, Nuclear Science and Technology Research Institute, Tehran 14155–1339, Iran; mmojtahedzadeh@aeoi.org.ir; 4 Department of Physics, Membrane Science and Technology Research Center, Prince of Songkla University, Songkhla 90110, Thailand; pikul.v@psu.ac.th; 5 Department of Chemistry, Faculty of Science, University of Qom, Qom 3716146611, Iran; mahmoudnasr81@gmail.com; 6 Regional Centre of Advanced Technologies and Materials, Palacky University, Šlechtitelů 27, 783 71 Olomouc, Czech Republic

**Keywords:** chitosan membrane, methane ion, surface modification, characterization, ATR-FTIR

## Abstract

Chitosan has been used for biomedical applications in recent years, primarily because of its biocompatibility. A chitosan membrane with a 30 μm thickness was prepared and investigated for its surface modification using methane ions. Methane ions were implanted into the chitosan membrane using a Kaufman ion source; bombardment was accomplished using three accelerating voltages of ion beams—30, 55, and 80 kV. The influence of the ion bombardment on morphology, crystallinity, and hydrophilicity was investigated. Attenuated total reflectance Fourier-transform infrared (ATR-FTIR) spectroscopy analysis showed that a triplet bond appeared after the implantation of methane ions (acceleration voltage: 80 kV), culminating in the creation of a more amorphous membrane structure. The analyses of atomic force microscopy (AFM) images showed that, with the increase in bombardment energy, the roughness of the surface changed. These results revealed that ion bombardment improved the hydrophilicity of the membranes and the water fluxes of chitosan membranes altered after methane ion bombardment.

## 1. Introduction

Chitosan is obtained from chitin by deacetylation and is an abundant polysaccharide usually produced from seafood as a waste product [1]. It has garnered significant notice because of its potential beneficial applications in medical science, especially in pharmaceutical areas, due to its biocompatibility, low toxicity, and biodegradability [2,3,4,5,6,7,8,9,10,11]. Yu and his colleagues [12] have shown that chitosan membranes after modification by ion beams represented the cell membrane mechanism during the transferring of genes. Researchers have been exploring the modification of polymers by plasmas, lasers, UV lamps, electron beams, ion beams, and gamma rays [13,14,15,16,17,18,19,20,21]. The ion beam implantation technique is a well-known and effective method to modify polymers [22,23]. This method can be used to modify the surface without affecting the bulk structure. Radiation treatment is a fast and relatively clean method and can alter physico-chemical properties of polymers due to chain scissoring, cross-linking, carbonization, oxidation, and radical formation [24,25,26,27,28,29] and does improve the electrical properties of ion exchange membranes [30,31]. These changes result in improvements in mechanical, electrical, and thermal properties of polymers, depending on the polymer type and irradiation [32].

This work is aimed to surmount the typical drawbacks of chitosan membranes. The main hindrance to use chitosan is its low hydrophilicity nature (although chitosan has hydrophilic hydroxyl groups and acetyl groups, it also has hydrophobic backbone), and it needs to be rendered more hydrophilic. In the present work, chitosan membranes were implanted by ions at different accelerating voltages of ion beams with the main purpose to explore the influence of methane ion implantation on the crystallinity and hydrophilicity of chitosan membranes. Natural gas is a proper substitute for crude oil, which might run out in the next century, as a feedstock in chemical and pharmaceutical industry. Since methane (CH_4_) is a cost-effective option and has a broad range of use, it is considered as the main component of natural gas. In this manuscript, the morphologies of the samples were investigated by field emission scanning electron microscopy (FESEM) and atomic force microscopy (AFM). According to the obtained results, the surface hydrophilicity of the membranes was increased, which led to an improvement in the membrane performances.

## 2. Results and Discussions

### 2.1. Theory

Methane gas is decomposed to fragments like H^+^, H_2_^+^, C^+^, CH^+^, CH_2_^+^, CH_3_^+^, and CH_4_^+^ as a consequence of tungsten cathode electron emission with each fragment accelerating towards chitosan membranes. In the case of the kinetic energies of these compounds supposed to be *E_0_* (30, 55, and 80 keV here), the hydrogen and carbon kinetic energies are assessed by the following Equations (1) and (2), respectively [33]:

(1) $$E_{H}=\left[\frac{m_{H}}{m_{c}+nm_{H}}\right]E_{0},$$

(2) $$E_{c}=\left[\frac{m_{c}}{m_{c}+nm_{H}}\right]E_{0}.$$

In as much as the binding energy between C and H (in order of eV) is thousands times less than the projectile energy, the entities break totally into C and H. This could happen, when they bump the surface of the membrane and each ion of C and H enters the chitosan membrane with nearly the identical kinetic energy as in the compound [33]. Stopping and Range of Ions in Matter (SRIM) is a group of computer programs, which calculate interaction of ions with matter; the core of SRIM is a program Transport of ions in matter (TRIM). SRIM is popular in the ion implantation research and technology community and is also used widely in other branches of radiation material science. SRIM is based on a Monte Carlo simulation method, namely the binary collision approximation with a random selection of the impact parameter of the next colliding ion [34,35,36]. The hydrogen (d_H_) and carbon (d_c_) projectile ranges were computed near close to the surface using SRIM 2008 (Table 1). The mass density and chemical structure of chitosan are 0.25 g cm^−3^ and C_6_H_11_O_4_N.

### 2.2. XRD

**Figure 1:**
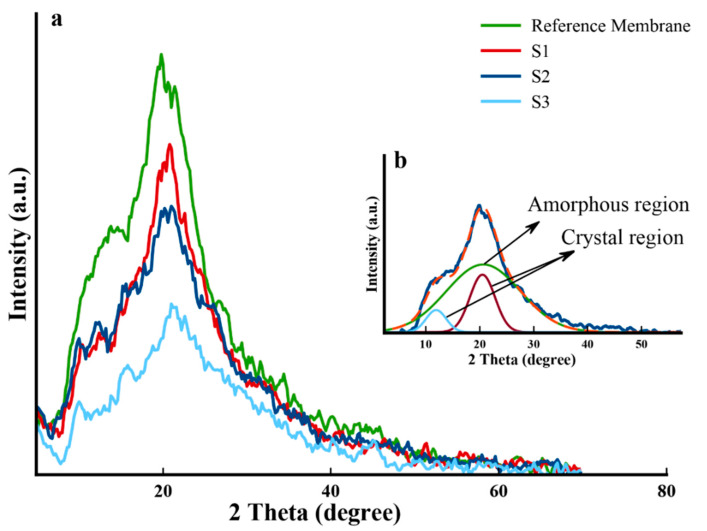
(**a**) XRD patterns of the reference membrane and the irradiated membrane. (**b**) Deconvolution of the XRD pattern of the reference sample.

The XRD measurement was performed to examine the ion bombardment effect on the crystallinity of the samples. The diffraction patterns of the membranes are depicted in Figure 1. The samples with different accelerating voltages of ion beams are shown with the names S1 (30 kV), S2 (50 kV), and S3 (80 kV), respectively.

The percentage of crystallinity can be estimated by deconvoluting each XRD peak with different methods. Some deconvolution and curve fitting methods were reported in Reference [37]. The percentage of crystallinity (%) was estimated via the area ratio of all crystalline peaks with respect to the entire area including noncrystalline fraction, as shown in Equation (3) [37]:

(3) $$Crystallinity\;\left(\%\right)=\left(\frac{A_{c}}{A_{c}+A_{a}}\right)\times 100\%,$$

where *A_c_* is the sum of all crystalline peaks areas and *A_a_* is the amorphous peak area. The peak fitting demonstrated an amorphous nature for chitosan. In order to calculate the percentage of the crystallinity of chitosan, three Gaussian peaks were fitted and were determined according to the XRD deconvolution method [37]. The two peaks fitted at ~11° and ~20° corresponded to the crystalline structure, while the shoulder fitted at ~21° appertained to the amorphous phase [38,39]. The obtained crystallinity values for all samples are listed in Table 2, which showed that the relative crystallinity amount of the membrane decreased from 27.7% to 17.3%. Thus, the irradiated samples became more amorphous than the reference membrane, probably due to the scissoring of these bonds and the local movement of free radicals [40].

### 2.3. Attenuated Total Reflectance Fourier-Transform Infrared (ATR-FTIR) Spectroscopy Characterization

**Figure 2:**
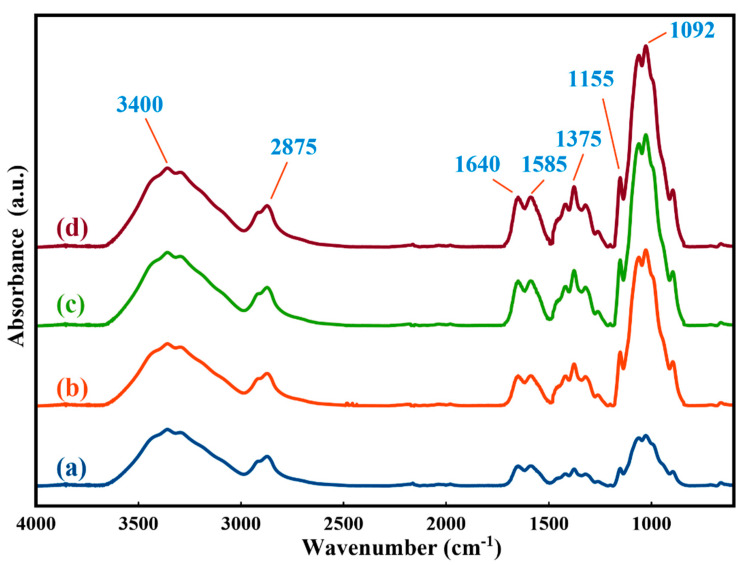
Attenuated total reflectance Fourier-transform infrared (ATR-FTIR) spectra of the reference sample (**a**), S1 (**b**), S2 (**c**), and S3 (**d**).

The ensuing structural functional group alterations were studied by ATR-FTIR spectroscopy. Figure 2 illustrates a comparison between the ATR-FTIR spectra of the irradiated samples and the spectrum of the reference sample; the major characteristic peaks of chitosan are listed in Table 3 [41,42,43]. In the ATR-FTIR spectra, the intensities of characteristic peaks at 3400, 2875, 1640, 1375, 1155, and 1092 cm^−1^ increased after ion irradiation. These results indicated that after ion bombardment, the intensities of the polar groups increased. Figure 2d indicates that C–H and C–C bonds were cleaved and chains were partially destroyed.

### 2.4. Surface Morphology Studies

**Figure 3:**
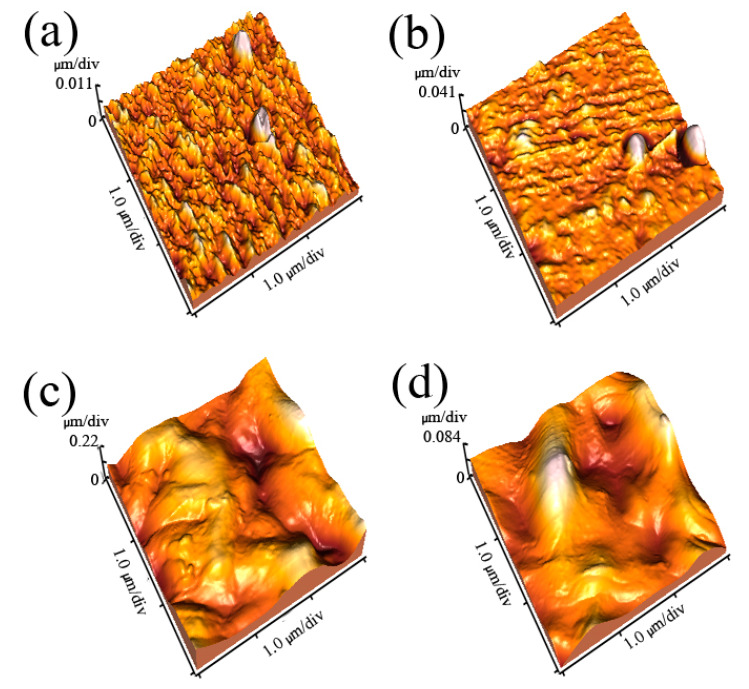
Atomic force microscopy (AFM) images of the reference membrane surface (**a**), S1 (**b**), S2 (**c**), and S3 (**d**).

**Figure 4:**
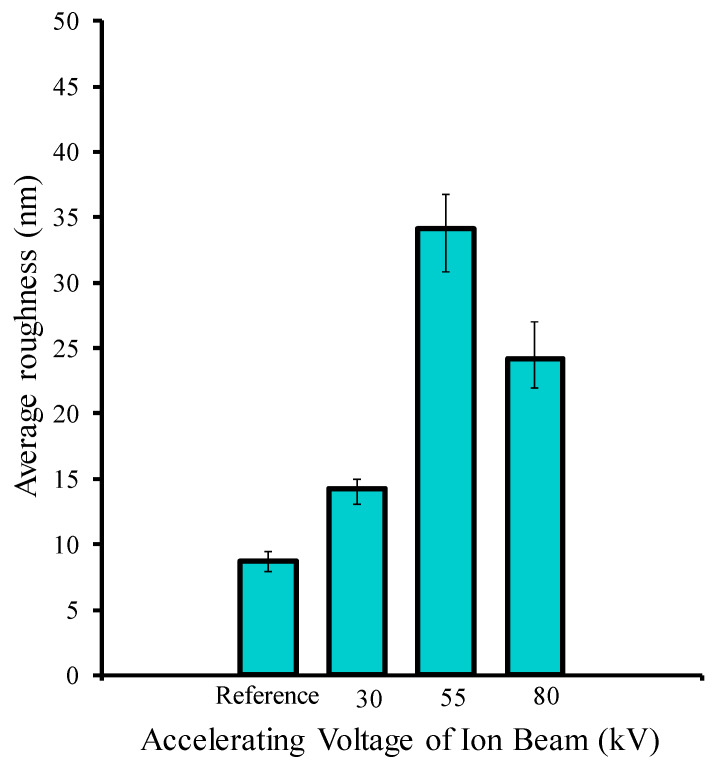
Surface average roughness of samples.

The morphological structures of the reference and irradiated chitosan membranes were studied by AFM as shown in Figure 3, with the details of AFM images for the reference and methane ion-bombarded chitosan, which rendered the surface rougher. The increase in roughness may be due to the methane ion ablation of the surface membrane. The average roughness is depicted in Figure 4. As can be seen, the roughness of the irradiated sample initially increased as compared to the reference sample and then decreased after being irradiated by ions with an accelerating voltage of 55 kV.

**Figure 5:**
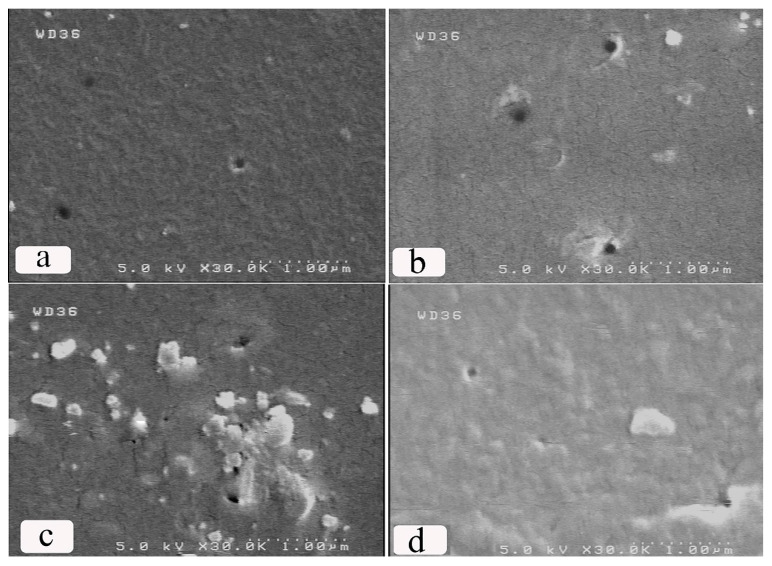
The field emission scanning electron microscopy (FESEM) micrographs of the reference sample (**a**), S1 (**b**), S2 (**c**), and S3 (**d**).

An alternative method of studying polymer surfaces is scanning electron microscopy. Figure 5 represents the FESEM micrographs of the reference and ion bombardment samples, which showed the increased pore sizes of the samples irradiated by ions with an accelerating voltage of 30 kV (Figure 5b). Ion implantation in the membranes induced deposited charges and probably electrostatic repulsion between them, and the inherent cationic charges of the membranes were responsible for the increased pore size. With the increase in the accelerating voltage of ion beams (ion beam with a higher energy) (Figure 5c,d), the surface of chitosan shrank slowly. Thus, the decrease in the pore size of the chitosan membrane may be due to the shrinkage of the surface membrane.

### 2.5. Contact Angle Measurements

**Figure 6:**
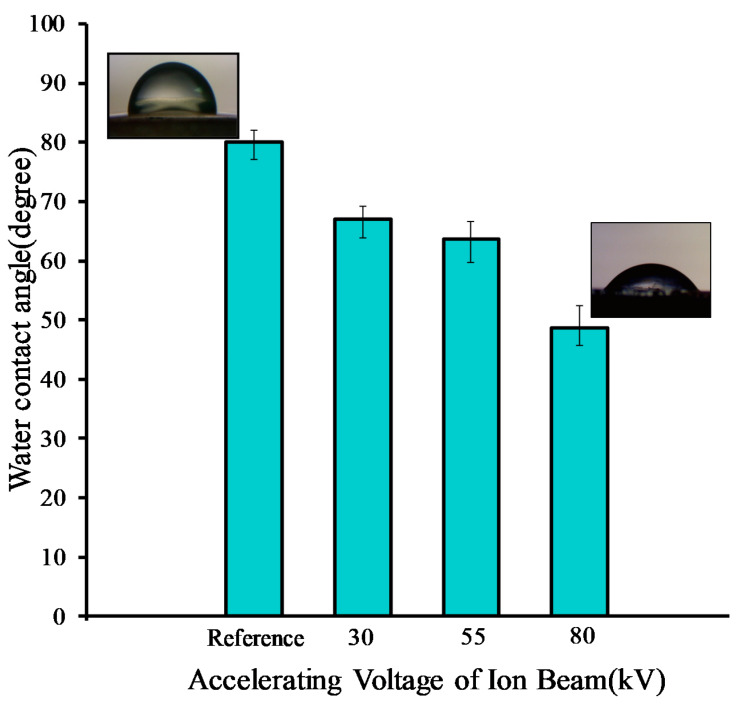
Comparison of the water contact angles of the chitosan membrane (reference sample) and the irradiated chitosan membrane at different accelerating voltages of ion beams.

Figure 6 shows water contact angles (WCAs) for the chitosan membrane before and after the irradiation; the WCA of the chitosan membrane decreased from 80° to 48° after ion bombardment with an accelerating voltage of 80 kV. Considering the reference value, the reduction in contact angle was almost 40%, which can be mainly attributed to the oxygenated functional groups, namely OH, C=O, etc. [44,45]. Moreover, the AFM affirmed the surface roughening of the membrane after ion methane bombardment. Water spread to a larger extent because of pores and tiny voids on the surface, to decrease the contact angle, where both functional groups and surface roughening played important roles [44,45].

The wettability of a solid surface is influenced by both its geometric structure and its chemical composition, and as the roughness of a surface increases, its WCA tends to increase or decrease, depending on whether the surface is hydrophobic or hydrophilic [46].

### 2.6. Water Flux Characterizations

**Figure 7:**
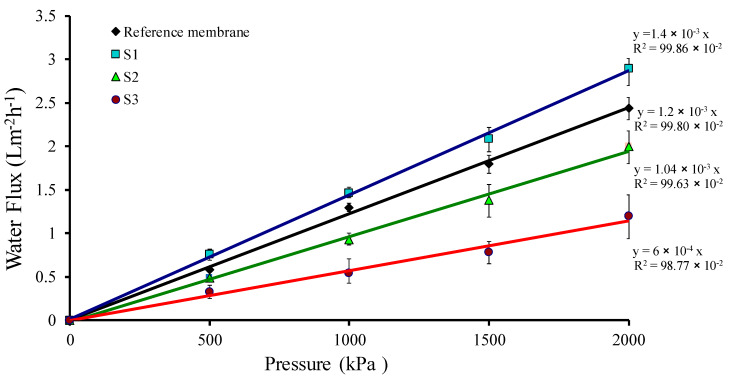
Water fluxes of the chitosan membrane (reference sample) and the irradiated chitosan membrane at different accelerating voltages of ion beams.

A piece of the membrane with a known area was placed in a dead-end filtration unit, and N_2_ gas was applied to the unit at varying pressures. A series of water flux (J) and its corresponding pressure (ΔP) were recorded after each period of time. The hydraulic permeability coefficient (L_p_) were acquired from the graph slope amongst the flux and the pressure used; the relation was shown as J = L_p_ ΔP, known as the Hagen–Poiseuille equation [47,48,49]. The water flux (J) across the membrane was predicted and plotted against the used pressure. Figure 7 displays the water flux of the irradiated membranes related to the reference ones and illustrates a linear relationship between the flux and the pressure used for the membranes. Leveneur et al. studied ion implantation and further chemical and structural changes mostly based on a ballistic process. According to the process, the fluence changes lead to different results. Increasing the fluence initiate the degradation of the cellulose and the degradation and breakage of the intermolecular bonds. During ion implantation, the energy carried by the incident ions is deposited within the implantation range. This initially results in high local and transient increase of temperature or thermal spikes. This energy is then transferred through heat conduction to the entire sample. On the other hand, electrostatic repulsion between the cationic fixed charges of the membrane and the deposited charge from the implanted methane ions might cause negligible increase in water flux of samples irradiated with an accelerating voltage of an ion beam (30 kV). The higher value of the severe decline of the water flux is due to the increase in the accelerating voltage of the ion beam, which results in the decrease of the porosity and pore size of the membrane surface. Figure 5 shows the FESEM micrographs of a pore sealant after ion bombardment with the increase in the accelerating voltage of the ion beam due to the shrinkage of the surface membrane. This influenced the whole pore area of the membrane. Table 4 shows hydraulic permeability (*L_p_*) values obtained from the slope of the graph in Figure 7, and the membranes were categorized as reverse osmosis ones [50].

## 3. Materials and Methods

### 3.1. Materials

Chitosan membranes were prepared by the solvent evaporation method following the preparation process described in detail by Wanichapichart [51]. The ensuing membranes were bombarded with methane ion beams.

### 3.2. Ion Beam Bombardment

The ion implantation technique was performed under vacuum conditions, where the vacuum chamber was drained under 2.6 × 10^−3^ Pa. With the aid of a Kaufman ion source under a 5 × 10^−3^ Pa working pressure (Chengdu, China), the methane ions were implanted into the sample via bombardment with methane ion beams utilizing three accelerating voltages—30, 55, and 80 kV. To avoid the destruction of the sample surface due to heat, a low current density of 40 μA cm^−2^ was chosen, for which a low injection of gas would be sufficient; the ion fluence and the exposure time in the implantation process were 10^16^ ions/cm^2^ and 1 min, respectively.

It is worth noting that at the base pressure of this experiment, oxygen and nitrogen as residual gases could affect the surfaces of the irradiated samples by bond-breaking and the formation of free radical, the breakage of the intermolecular bonds near the surface, and the recombination of polymers on neighboring surfaces [22]. However, comparing the amounts of oxygen and nitrogen with that of methane, their effects were considered negligible.

### 3.3. Methods and Characterizations

The membranes were irradiated with methane ions for modification purposes. The crystallinity of the reference (bare membrane) and the irradiated membranes were studied via XRD by deploying a Philips powder diffractometer, type PW 1373 goniometer (Philips, Amsterdam, The Netherlands). Functional groups of polymers were ascertained using ATR-FTIR (Bruker Alpha, Yokohama, Japan). Likewise, the surface structures of the membranes were probed using AFM Veeco Autoprobe CP Research (Veeco, Plainview, NY, USA) and FESEM Hitachi model S-4160 (Hitachi, Tokyo, Japan). The evaluation of the roughness parameter of the sample was based on a scanned area of 3 µm × 3 µm. The WCA measurement is typically used to acquire the relative hydrophilicity of a polymer membrane surface. After the irradiation of membranes, to convince that any variation in flux measurements was completely autonomous of this water swelling property for at least 30 min, all membrane samples were immersed in distilled water. In order to gauge the permeating water volume under numerous applied pressures, a dead-end filtration unit was utilized.

## 4. Conclusions

In this work, we reported the effect of CH_4_ ion bombardment on structural and morphological characteristics of a chitosan membrane. The XRD spectra showed that the irradiated samples became more amorphous with respect to the reference membrane, which may be attributed to the increase in temperature at the surface of the samples and the cleavage of the bonds, thus enhancing the disorder in the chitosan structure. The increasing polar functional groups and alterations in the surface roughness were responsible for enhancing the wettability of the chitosan membranes. Thus, with the help of low-energy methane ion beam bombardment, the filtering characteristics of chitosan membranes reverse osmosis could be adjusted.

## Figures and Tables

**Table 1:** The projectiles of carbon (d_c_) and hydrogen (d_H_) by SRIM 2008.

	E_0_ (30 keV)				E_0_ (55 keV)				E_0_ (80 keV)			
	E_c_	E_H_	d_c_	d_H_	E_c_	E_H_	d_c_	d_H_	E_c_	E_H_	d_c_	d_c_
	(keV)	(keV)	(Å)		(keV)	(keV)			(keV)	(keV)	(µm)	
**CH_4_**	22.5	1.875	4999	2460 Å	41.25	3.428	8935 Å	4220 Å	60	5	1.27	5807 Å
**CH_3_**	24	2	5320	2680 Å	44	3.66	9499 Å	4455 Å	64	5.33	1.35	6124 Å
**CH_2_**	25.7	2.14	5683	2772 Å	47.14	3.93	1.01 µm	4737 Å	68.57	5.71	1.44	6483 Å
**CH**	27.7	2.3	6108	2958 Å	50.77	4.23	1.09 µm	5077 Å	73.85	6.15	1.54	6889 Å
**C**	30		6594		55	4.23	1.17 µm		80		1.66	
**H_2_**		15		1.37 µm		27.5		2.12 µm		40		2.77 µm
**H**		30		2.26 µm		55		3.49 µm		80		4.65 µm

**Table 2:** Percentage of crystallinity.

Membrane	Crystallinity (%)
Reference membrane	27.7
S1	25.9
S2	22.4
S3	17.3

**Table 3:** Bands and characteristic peaks of chitosan.

Band	Vibrations Peak (cm^−1^)
(O–H stretch)	3400
(C–H stretch)	2875
(C=O stretch, amide group)	1640
(N–H deformation, amino group)	1585
(C–O stretch, amide group)	1375
(bridge O stretch)	1155
(C–O stretch)	1092

**Table 4:** Hydraulic permeability of samples.

Sample	L_p_ × 10^−13^ (m^3^ N^−1^ S^−1^)
Reference membrane	3.33
S1	3.89
S2	2.89
S3	1.67
